# The Impact of Surgical Trauma-Activated Platelet-Rich Fibrin on Mesenchymal Stromal Cells In Vitro

**DOI:** 10.3390/cells15100945

**Published:** 2026-05-21

**Authors:** René D. Verboket, Lea Usov, Isabell Bohl, Jonas Neijhoft, Marissa Penna-Martinez, Ingo Marzi, Dirk Henrich

**Affiliations:** Department of Trauma Surgery and Orthopaedics, University Hospital, Goethe University Frankfurt, 60590 Frankfurt, Germany

**Keywords:** bone regeneration, PRF, tissue regeneration, trauma, platelets, tissue engineering

## Abstract

Introduction: platelet-rich fibrin (PRF) is a second-generation platelet concentrate which is known for promoting cell migration, tissue repair, angiogenesis and bone formation. In contrast, the specific effects of trauma-activated PRF on mesenchymal stromal cells (MSC) are not yet fully understood. The present study investigates systemic effects of surgical trauma-activated PRF on MSCs in vitro, analyzing their metabolic activity, inflammatory responses, and regenerative capacity to optimize advanced treatment concepts for severe fractures and injuries. Material & Methods: PRF membranes (T-PRF from trauma patients, C-PRF from healthy controls) were generated. After co-incubation with MSC cells for 24, 72, and 120 h, further investigations of metabolic activity (MTT assay) and gene expression analyses were performed. Results: for MTT assay, results especially showed a significantly higher metabolic activity of T-PRF after 120 h. ELISA-results measuring cytokine levels (CXCL10, IL-6, VEGF, and IDO) exposed a frequent peak in T-PRF group at 72 h, declining slightly at 120 h. In the gene expression analyses, T-PRF exerted a comparatively stronger stimulating effect on MAPK14 and VEGFA after 24 h, while a decrease in gene expression for MAPK8, MAPK14, and RUNX2 was observed over time. Conclusion: surgical trauma-activated PRF seems to be a powerful inducer of early inflammatory and stress responses in MSCs with preserved angiogenic but limited osteogenic signaling. Therefore, a targeted balance between inflammatory activation and sustainable regeneration, as well as optimized preparation and possible combination with immunomodulatory approaches, appear to be crucial for the therapeutic success of PRF-based strategies.

## 1. Introduction

Platelet-rich fibrin (PRF) has emerged as a key biomaterial in regenerative medicine due to its natural, autologous nature and its ability to release a variety of bioactive molecules that promote tissue healing [1]. As a second-generation platelet concentrate, PRF is produced by centrifuging whole blood to separate its components [2]. Unlike platelet-rich plasma (PRP), PRF contains a fibrin matrix that forms during the centrifugation process which stabilizes the growth factors and cytokines it releases, providing prolonged biological activity in the healing process [3,4,5]. PRF has been extensively used in dental, orthopedic, and wound healing applications, where it is applied to accelerate tissue regeneration by promoting cell migration, angiogenesis, and matrix synthesis [6,7].

### 1.1. The Role of Platelet-Rich Fibrin in Regenerative Medicine

Platelet-rich fibrin is created by centrifuging whole blood at specific speeds and time intervals to separate the blood’s components [8]. The process leads to the formation of a fibrin clot enriched with platelets, leukocytes, and growth factors such as vascular endothelial growth factor (VEGF), platelet-derived growth factor (PDGF), and transforming growth factor beta (TGF-β) [9,10]. This fibrin clot, once applied to an injury site, is believed to act as a scaffold for cell migration and tissue regeneration, as well as a reservoir for the sustained release of growth factors [11]. PRF’s ability to accelerate wound healing and support bone regeneration has made it a powerful tool in various clinical settings [12]. For example, PRF has been widely applied in periodontal and regenerative procedures. It was demonstrated that the combination of leukocyte- and platelet-rich fibrin with autogenous bone grafts yields outcomes comparable to established techniques using collagen membranes in the treatment of intrabony defects [13].

The biological effects of PRF have been shown to include increased angiogenesis, enhanced tissue remodeling, and the promotion of osteogenesis. In bone regeneration, PRF facilitates the recruitment of stem cells, enhances the differentiation of osteoblasts, and accelerates the deposition of extracellular matrix, contributing to the formation of new bone tissue [14,15]. Furthermore, PRF’s ability to support wound healing also includes its role in modulating the inflammatory response. The inflammatory phase of wound healing is essential for the initial defense against pathogens and the initiation of repair processes, but prolonged or excessive inflammation can impair healing. PRF’s ability to balance this inflammatory phase and promote a shift towards the proliferative and remodeling phases is one of the reasons for its success in regenerative applications [16,17].

Despite these promising applications, the inflammatory properties of PRF under unphysiological conditions remain underexplored. The influence of trauma on the composition of PRF and its subsequent effects on stem cell activity could have profound implications for its clinical application [18,19]. Trauma-induced changes in the inflammatory environment, such as the release of cytokines, immune cell activation, and altered blood flow, may impact the regenerative potential of MSCs and other stem cells [20,21]. Furthermore, trauma often results in tissue defects that are potential candidates for PRF application. This understanding is particularly crucial when considering the use of PRF in acute settings, where inflammation is often heightened due to the injury.

Previous research demonstrated that A-PRF from trauma patients had a higher ratio of inflammatory to anti-inflammatory macrophages, and it is conceivable that the 3D fibrin matrix formed by the PRF provides additional stimulatory signals to the embedded cells [19,22].

### 1.2. Mesenchymal Stromal Cells and Their Role in Regeneration

Mesenchymal stromal cells (MSCs) are multipotent stromal cells that play a key role in tissue regeneration, primarily by differentiating into various cell types, including osteoblasts, chondrocytes, and adipocytes [23,24]. These cells are present in various tissues, including bone marrow, adipose tissue, and the synovial membrane, and are known for their ability to migrate to injured areas, where they contribute to tissue repair [25,26,27]. In addition to their differentiation potential, MSCs possess anti-inflammatory properties, modulate immune responses, and secrete growth factors that further enhance tissue healing [28,29,30,31].

MSCs are a critical component of bone and cartilage regeneration, where their ability to differentiate into osteoblasts and chondrocytes is vital for the formation of new tissue. In bone repair, MSCs migrate to the site of injury, where they differentiate into osteoblasts, the cells responsible for bone formation [32]. This regenerative process is influenced by a variety of factors, including the local microenvironment, which can be altered by biomaterials such as PRF. The interaction between MSCs and PRF has been shown to support cell migration, proliferation, and differentiation, making MSCs and PRF a potentially powerful combination for tissue repair [33,34].

In addition to their regenerative capacity, MSCs are capable of modulating the immune response [35,36]. By secreting immunomodulatory molecules, MSCs can shift the balance from pro-inflammatory to anti-inflammatory signaling, potentially preventing chronic inflammation that would otherwise impair healing [37]. However, the interaction between trauma-activated PRF and MSCs could alter these immune-modulating effects.

### 1.3. Trauma-Activated Platelet-Rich Fibrin: A Unique Profile

Trauma-activated PRF differs from standard PRF in its origin. It is prepared from blood collected from trauma patients, who typically exhibit an acute inflammatory state characterized by elevated levels of circulating cytokines, growth factors, and other immune modulators [19]. Trauma induces the activation of platelets and the release of pro-inflammatory cytokines such as interleukin (IL)-6, tumor necrosis factor-alpha (TNF-α), and IL-1β [38]. These inflammatory mediators are essential for initiating the repair process by promoting immune cell recruitment and tissue protection, but they may also alter the behavior of stromal cells and other regenerative cells, either facilitating or impeding healing.

While PRF’s ability to support tissue regeneration has been well-documented, the unique inflammatory profile of trauma-activated PRF presents a challenge. On the one hand, the inflammation induced by trauma-activated PRF may enhance the migration of MSCs to the injury site, accelerate tissue repair, and promote the activation of regenerative processes [39]. On the other hand, excessive inflammation may impede these regenerative processes, as chronic inflammation is known to suppress stem cell differentiation and prolong the inflammatory phase of healing [40]. Furthermore, the trauma-associated cytokine release may interfere with the regenerative potential of PRF itself, altering its capacity to support stem cell differentiation and tissue regeneration.

The exact mechanisms by which trauma-activated PRF affects MSC behavior are not yet fully understood. Previous studies have shown that the presence of inflammatory cytokines in the microenvironment can modulate MSC differentiation pathways, but the effects of trauma-induced inflammation on MSCs in the presence of PRF have not been systematically explored [41,42]. This gap in knowledge forms the basis of this study, which seeks to clarify the relationship between trauma-activated PRF and MSC activity and to explore how this interaction influences the balance between inflammation and regeneration.

Therefore, it is essential to explore how trauma-activated PRF affects MSCs in both the short and long term, considering both inflammatory and regenerative responses.

### 1.4. Aims of the Study

The aim of this exploratory study is to investigate the putative effects of trauma-activated PRF on mesenchymal stromal cells in vitro, focusing on their metabolic activity, inflammatory responses, and regenerative capacity. This study bridges the gap between two important components in regenerative medicine: PRF and MSCs. While both PRF and MSCs have been widely studied individually, their interaction in the context of trauma-induced inflammation has not been thoroughly investigated. The findings from this study may offer insights into how surgical trauma-activated PRF can be used more effectively in clinical settings, particularly for patients with acute injuries or complex fractures. Furthermore, this research may lead to the development of new strategies to modulate PRF preparations to optimize their regenerative potential and minimize any adverse inflammatory effects.

## 2. Materials and Methods

### 2.1. Ethics

This study was conducted in accordance with the Declaration of Helsinki and approved by the local ethics committee of Goethe University Frankfurt. Ethical approval for the isolation and use of mesenchymal stromal cells (MSCs) was obtained under protocol no. 329/10, and approval for platelet-rich fibrin (PRF) generation was granted under protocol no. 68/17. Written informed consent was obtained from all participants prior to inclusion.

### 2.2. Study Concept

**Figure 1:**
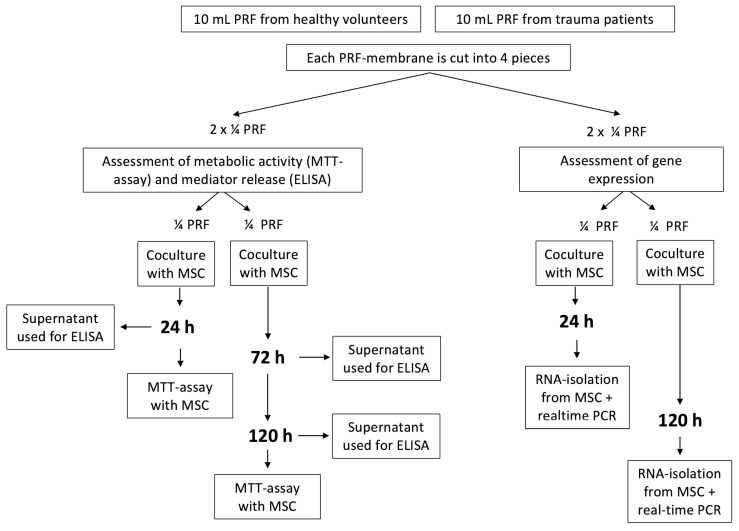
Study protocol, sampling, processing and measurements.

An in vitro coculture model was established to investigate the effects of surgical trauma-activated platelet-rich fibrin compared with PRF derived from healthy volunteers on MSCs. MSC metabolic activity, mediator release in coculture supernatants, and MSC gene expression were assessed at defined time points (Figure 1).

Venous blood samples were collected from patients with a single trauma of the extremities one day after surgical treatment to capture surgical trauma-associated inflammatory profiles. Age-matched healthy volunteers served as control. For all participants, 10 mL of venous blood was drawn into glass-coated A-PRF+ vacuum tubes (Process for PRF, Nice, France) to allow natural coagulation and subsequently centrifuged at 1300 rpm (208× *g*) for 8 min using an A-PRF Duo centrifuge. PRF clots were immediately harvested, and the fibrin-rich portion was isolated.

To ensure standardized processing, PRF clots were gently compressed into membranes using sterile instruments and subsequently subdivided into four equally sized pieces for reproducible application in coculture experiments.

**Figure 2:**
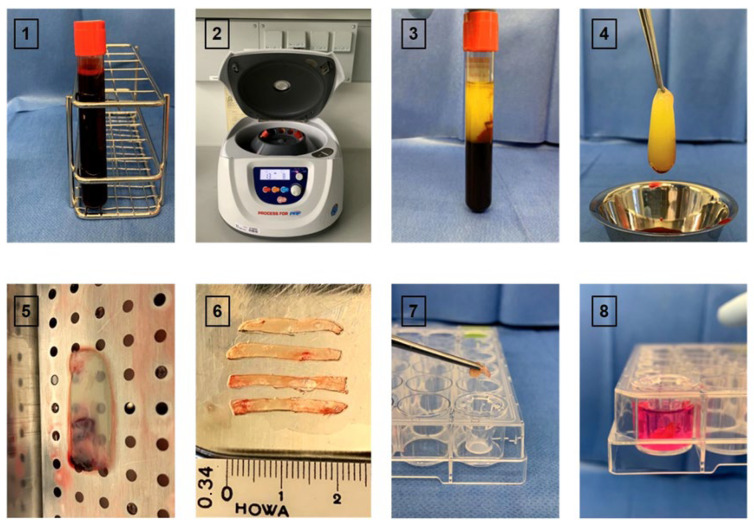
Preparation of platelet-rich fibrin (PRF) membranes. Schematic illustration of the individual steps involved in PRF preparation. After venous blood collection (**1**), samples were centrifuged using the A-PRF Duo centrifuge (**2**). The resulting PRF clot was removed using sterile forceps (**3**,**4**) and gently compressed on a sterile plate to form a membrane (**5**). Each membrane was longitudinally divided with a scalpel into four equally sized pieces (**6**). Individual PRF pieces were placed into transwell inserts and used for subsequent analyses (**7**). Transwells were filled with culture medium, fully covering the PRF membranes (**8**).

For each donor, PRF membranes were subdivided into four equally sized parts (Figure 2), which were allocated to different experimental analyses. Care was taken to ensure a comparable cellular composition across all fragments by standardized sectioning of the membranes. Each donor was considered the independent biological unit (n = 7). PRF fragments derived from the same donor were not treated as independent replicates within a given analysis but were instead used for distinct experimental readouts. This approach was chosen to avoid pseudoreplication. Throughout the manuscript, PRF derived from healthy volunteers is referred to as control PRF (C-PRF), whereas PRF obtained from surgical trauma patients is termed trauma-activated PRF (T-PRF).

### 2.3. MSC Isolation, Culture, and Characterization

Cryopreserved pooled human MSCs were used for all experiments. The MSC pool consisted of cells derived from bone marrow aspirates of five healthy donors and had been previously described and comprehensively characterized [43]. The use of residual bone marrow material for research purposes was approved under ethics protocol no. 329/10. All donors provided written informed consent.

MSCs were cryopreserved after passage four [43]. For experiments, pooled MSCs were thawed and seeded at a density of 1 × 10^5^ cells/cm^2^ in T25 flasks. Cells were expanded for two additional passages at 37 °C in a humidified atmosphere containing 5% CO_2_ using MesenCult medium supplemented with 10% MesenCult stimulatory supplements (Stemcell Technologies, Cologne, Germany), hereafter referred to as “medium.” MSCs were passaged at approximately 80% confluency.

The MSCs displayed a typical immunophenotype (CD90^+^, CD105^+^, CD34^−^, CD45^−^) and retained the capacity for trilineage differentiation into osteogenic, adipogenic, and chondrogenic lineages (Figure S1), as previously reported [43].

### 2.4. MSC–PRF Coculture

**Figure 3:**
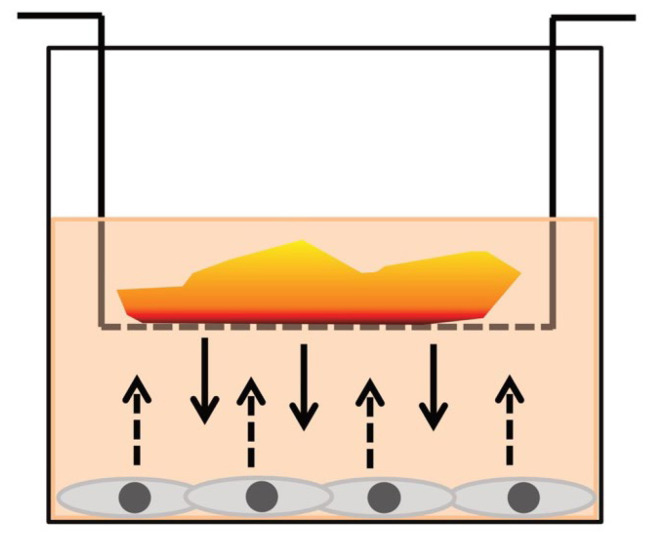
Schematic representation of the A-PRF–MSC coculture system. One representative well of a 24-well plate is depicted. Mesenchymal stromal cells (MSCs) are cultured on the bottom of the well in standard culture medium. A transwell insert containing a PRF membrane is placed above the MSC layer. Arrows indicate the bidirectional exchange of soluble mediators between PRF and MSCs.

For coculture experiments, MSCs were seeded into 24-well plates at a density of 2 × 10^4^ cells per well and allowed to adhere for 24 h. To assess the effects of PRF on MSCs, an indirect coculture approach was employed. Individual PRF membrane pieces (one per well), derived either from trauma patients or healthy controls, were placed into transwell inserts (pore size 0.4 µm; Corning, New York, NY, USA) and added to the MSC-containing wells. This setup allowed reciprocal exchange of soluble mediators between PRF-resident cells and MSCs without direct cell–cell contact (Figure 3).

MSC–PRF cocultures were maintained at 37 °C and 5% CO_2_ for 24 h or 120 h. Each PRF membrane was divided into four equal pieces (Figure 2), which were allocated as follows: one piece for assessment of MSC metabolic activity using the MTT assay, one piece for quantification of soluble mediators in culture supernatants by ELISA, and two pieces for analysis of PRF-induced effects on MSC differentiation and inflammatory responses by quantitative RT-PCR to increase amount of RNA for gene expression analysis.

### 2.5. MTT-Assay

MSC metabolic activity was assessed after 24 h and 120 h of coculture using the MTT assay (Hoffmann-La Roche, Basel, Switzerland) according to the manufacturer’s instructions. Transwell inserts containing PRF membranes were removed, and MSCs were incubated with MTT solution (30 µL MTT labeling reagent in MSC culture medium; total volume 300 µL) for 4 h at 37 °C. Formazan crystals generated by metabolically active cells were solubilized using the provided solubilization buffer, and absorbance was measured at 570 nm using a microplate reader (Infinite M200, Tecan, Männedorf, Switzerland).

### 2.6. Determination of Mediator Release with ELISA

Cytokine concentrations in coculture supernatants were determined using enzyme-linked immunosorbent assays (ELISA). Supernatants were collected after 24 h, 72 h, and 120 h of coculture. In each case, the culture medium was replaced 24 h prior to sampling to standardize the collection interval.

Collected supernatants were centrifuged at 300× *g* for 5 min to remove cellular debris and stored at −80 °C until analysis. Commercial ELISA kits were used to quantify IL-6, CXCL10, VEGF, and IDO-1 according to the manufacturers’ instructions (all kits from R&D Systems, Minneapolis, MN, USA). Absorbance was measured at 450 nm using a microplate reader (Infinite M200, Tecan, Männedorf, Switzerland).

### 2.7. Assessment of Differentiation and Inflammatory Gene Expression

To assess PRF-induced changes in MSC gene expression, total RNA was isolated using the RNeasy Mini Kit (Qiagen, Hilden, Germany) with on-column DNase treatment to eliminate genomic DNA contamination. RNA concentration and purity were determined using a NanoVue spectrophotometer (NanoDrop Technologies, Wilmington, DE, USA). Complementary DNA (cDNA) was synthesized from 500 ng total RNA using the AffinityScript cDNA Synthesis Kit (Agilent Technologies, Waldbronn, Germany).

Quantitative real-time PCR (qPCR) was performed using SYBR Green Master Mix on a CFX96 real-time PCR system (C1000 Touch, Bio-Rad, Dreieich, Germany). Genes associated with inflammation (IL6, CXCL10, MAPK8, MAPK14), anti-inflammatory responses (IDO1, TNFAIP6), angiogenesis (VEGFA), and osteogenic differentiation (RUNX2, COL1A1) were analyzed (Table 1). Relative gene expression was calculated using the 2^−ΔCt^ method and normalized to glyceraldehyde-3-phosphate dehydrogenase (GAPDH) as the housekeeping gene. All primers were obtained from Qiagen.

### 2.8. Statistics

The sample size was estimated based on prior experiments from our research group using a comparable MSC model system [43]. Given the exploratory nature of this study, the statistical analysis should be interpreted as hypothesis-generating.

Data are presented as boxplots of the median; whisker indicate minimum and maximum. Data distribution was assessed visually using residual plots. No extreme outliers were detected. Differences between groups were analyzed using a two-way repeated measures analysis of variance (ANOVA), with treatment (PRF vs. control) and time (24 h, 72 h, 120 h) as factors. Donor variability (n = 7) was accounted for by matching repeated measurements within each donor. The Geisser–Greenhouse correction was applied to adjust for potential violations of sphericity. Where appropriate, Tuckey‘s multiple comparisons test was used for post hoc analysis to compare (i) time points within each treatment group and (ii) PRF versus control at each time point. Statistical analysis was performed using GraphPad Prism 11 (GraphPad Software, San Diego, CA, USA). A *p*-value < 0.05 was considered statistically significant and *p*-values between 0.05 and 0.1 were judged as a statistical trend [44].

## 3. Results

The results of this study revealed significant effects of surgical trauma-activated PRF on the behavior of mesenchymal stromal cells (MSCs) over time, particularly in terms of cytokine production, metabolic activity, and gene expression.

PRF was obtained from seven patients with single trauma to the extremities on the first day post-surgery and compared with PRF from seven healthy volunteers. Both groups did not differ significantly regarding age (*p* = 0.81), and the sex distribution is comparable between both groups (Table 2). The mean time between trauma and surgery was 8.8 ± 4.3 days (range 4–15 days).

### 3.1. Surgical Trauma-Activated PRF: Dynamic Alteration of Metabolic Activity

The metabolic activity of MSCs was assessed using the MTT assay, which demonstrated dynamic responses to PRF exposure. Overall, incubation with PRF did not result in an increase in the metabolic activity of MSCs compared to the medium control. In addition, across all experimental groups, metabolic activity values at 120 h were higher than the corresponding values at 24 h. This increase was significant for the medium control and T-PRF.

**Figure 4:**
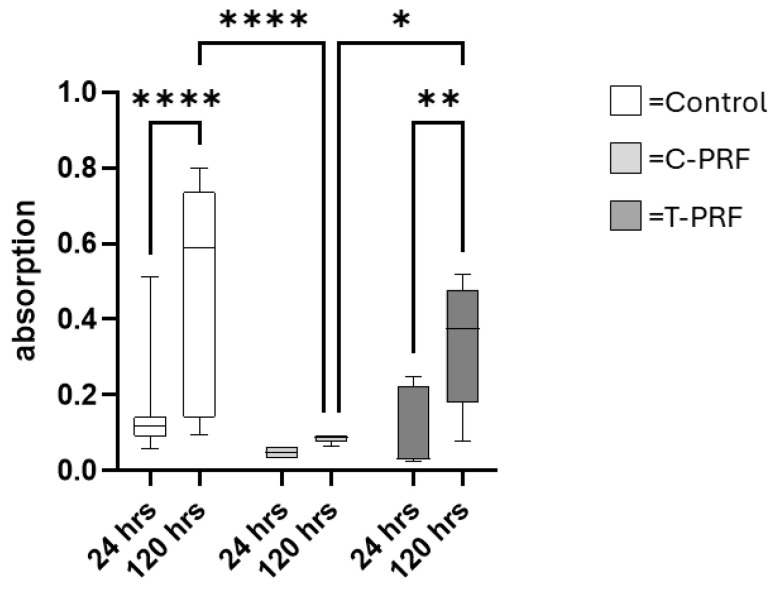
C-PRF incubation reduces MSC metabolic activity. Metabolic activity of MSCs measured by MTT assay following incubation with medium control (Con, white boxes), PRF from healthy volunteers (C-PRF, light grey boxes), or PRF from surgical trauma patients (T-PRF, dark grey boxes). Comparison between groups and time points (24 h and 120 h of incubation). Data are presented as box plots showing median values with minimum and maximum whiskers. * *p* < 0.05, ** *p* < 0.01, **** *p* < 0.001.

At the 24 h time point, the metabolic activity of MSCs in the medium control was higher compared with the C-PRF and T-PRF groups but without reaching statistical significance. After 120 h of incubation, metabolic activity in the T-PRF group was significantly higher than in the C-PRF group and reached approximately 65% of the value observed in the medium control (Figure 4).

### 3.2. Mediator Release Dependent from PRF-Source

To evaluate the development of mediator environment induced by PRF and MSC cocultures, cytokine levels in the culture supernatants were analyzed using ELISA after 24 h, 72 h and 120 h.

Overall, the lowest concentrations of the investigated mediators CXCL10, IL-6, VEGF, and IDO were measured mostly at 24 h. Subsequent temporal patterns differed between C-PRF and T-PRF. In C-PRF, a generally continuous increase in mediator concentrations was observed over 120 h, whereas in T-PRF a peak was frequently detected at 72 h, followed by a slight decline at 120 h.

CXCL10 is a mediator that can be released both by leukocytes within PRF and by inflammatory-stimulated MSCs. CXCL10 primarily mediates the recruitment of CD8^+^ T cells, Th1 cells, and NK cells and plays an important role in linking inflammation and tissue repair. In the PRF–MSC coculture, CXCL10 was the only mediator that increased in trend (*p* = 0.09) over time in both groups. However, no differences between the C-PRF and T-PRF approaches were observed at any of the respective time points.

Interleukin-6 is a pleiotropic cytokine with both pro-inflammatory and regeneration-promoting properties. It can be produced by MSCs as well as by leukocytes within PRF. At 72 h, IL-6 concentrations showed significantly higher levels in T-PRF compared with C-PRF, whereas at 120 h, values were in trend higher in the C-PRF group (*p* = 0.07).

The primary source of IDO-1 in the PRF–MSC coculture is presumably MSCs, although it can also be released to a lesser extent by leukocytes within PRF. IDO-1 exerts T-cell suppressive effects and promotes M2 macrophage polarization, thereby creating a microenvironment that supports tissue regeneration. IDO-1 concentrations increased significantly over time in C-PRF group (*p* < 0.05) and by trend in T-PRF (*p* < 0.1). At 72 h, IDO-1 levels were significantly higher in the T-PRF group compared with the C-PRF group (*p* < 0.05).

**Figure 5:**
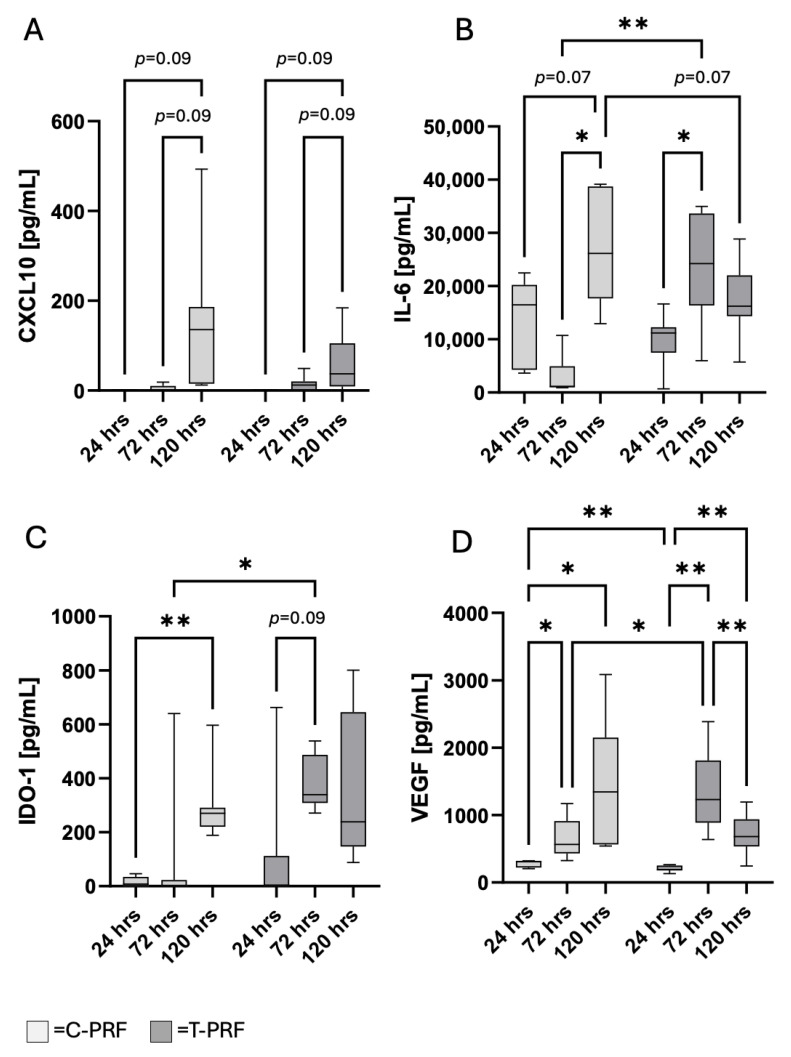
Time-dependent mediator release in PRF–MSC cocultures. Concentrations of CXCL10 (**A**), IL-6 (**B**), IDO-1 (**C**), and VEGF (**D**) in supernatants of cocultures containing control PRF (C-PRF) or surgical trauma-activated PRF (T-PRF) after 24 h, 72 h, and 120 h of incubation. For each time point, the collection phase lasted 24 h prior to sampling. Data are presented as box plots showing median values with minimum and maximum whiskers. Light grey boxes represent C-PRF, dark grey boxes represent T-PRF. * *p* < 0.05, ** *p* < 0.01.

In the PRF–MSC coculture, VEGF can be released by platelets and monocytes within PRF as well as by MSCs and primarily exerts pro-angiogenic effects. VEGF concentrations increased significantly over time in the C-PRF approaches, whereas a maximum was observed at 72 h in the T-PRF group. VEGF levels were significantly higher at 24 h and significantly lower at 72 h in C-PRF group compared with the corresponding time points in the T-PRF group (*p* < 0.05, Figure 5).

### 3.3. Differential Gene Expression Responses Induced by T-PRF

Genes associated with intracellular signaling and activation (MAPK8, MAPK14), inflammation (IL6, CXCL10), anti-inflammatory responses (IDO1, TNFAIP6), vascularization (VEGFA), and osteogenic differentiation (COL1A, RUNX2,) were analyzed.

Compared with the medium control, pronounced changes in gene expression were observed following treatment with C-PRF and/or T-PRF for MAPK8, MAPK14, IL6, VEGFA, RUNX2, and COL1A. These expression changes did not follow a consistent overall pattern. PRF treatment resulted in reduced expression of MAPK8 and RUNX2 genes (C-PRF and T-PRF at 120 h) as well as COL1A (C-PRF and T-PRF at 24 h), while increased expression levels were detected for IL6 and VEGFA (C-PRF and T-PRF at 24 h, *p* = 0.09, *p* < 0.05) and for MAPK14 (T-PRF at 24 h, *p* < 0.05).

**Figure 6:**
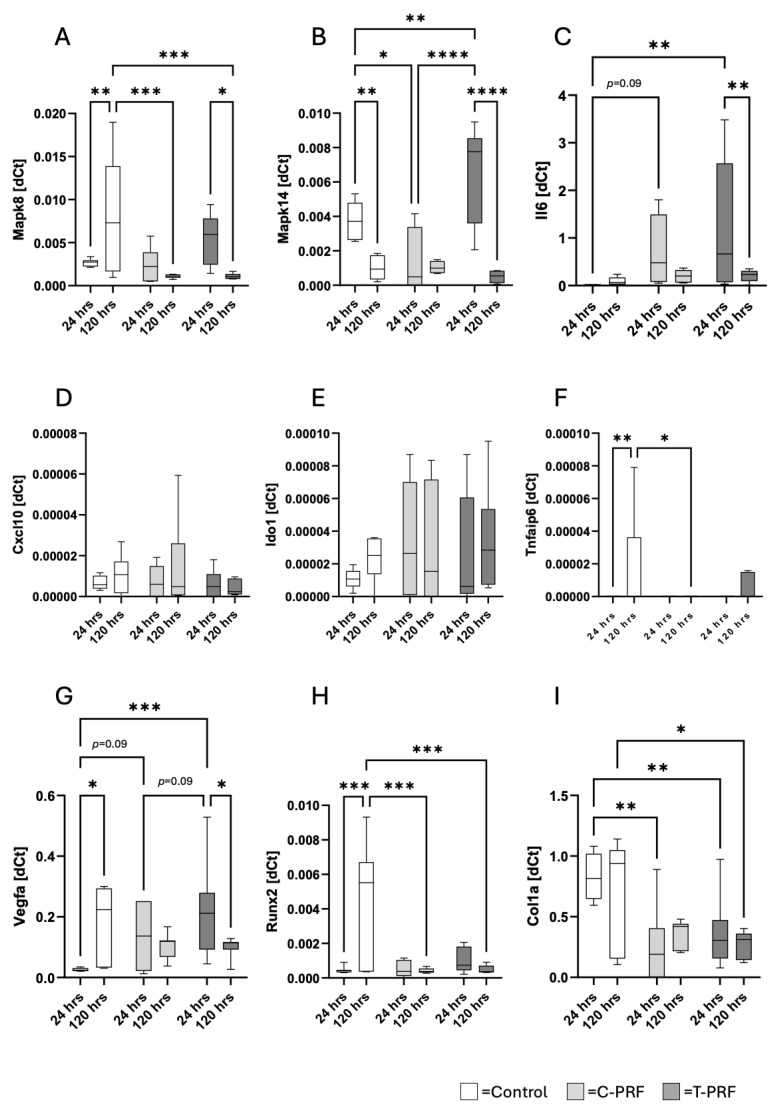
PRF-induced changes in MSC gene expression. Relative gene expression levels of MAPK8 (**A**), MAPK14 (**B**), IL-6 (**C**), CXCL10 (**D**), IDO-1 (**E**), TNFAIP6 (TSG-6, (**F**)), VEGFA (**G**), RUNX2 (**H**), and COL1A1 (**I**) in MSCs following incubation with C-PRF or T-PRF for 24 h and 120 h. Gene expression was normalized to GAPDH and calculated using the 2^−ΔCt^ method. Data are presented as box plots showing median values with minimum and maximum whiskers. White boxes represent medium control, light grey boxes represent C-PRF and dark grey boxes represent T-PRF. * *p* < 0.05, ** *p* < 0.01, *** *p* < 0.005, **** *p* < 0.001.

Furthermore, T-PRF at 24 h exerted a stronger stimulatory effect on MAPK14 (*p* < 0.05) and VEGFA gene expression (*p* = 0.09) compared with C-PRF at the same time point. Over time, a decrease in gene expression was observed for MAPK8 (T-PRF) and MAPK14 (T-PRF). RUNX2 gene expression was only slightly reduced (T-PRF, not significant), respectively, equally expressed (C-PRF) between both time points (Figure 6).

## 4. Discussion

This study provides a focused analysis of the effects of surgical trauma-activated platelet-rich fibrin (PRF) on mesenchymal stromal cells (MSCs), with particular emphasis on inflammatory activation versus regenerative potential. The data reveal a dual and temporally distinct role of trauma-activated PRF: an early induction of inflammatory and stress-related responses in MSCs, followed by partial metabolic and functional adaptation that probably does not translate into sustained osteogenic differentiation. These findings highlight the delicate balance between inflammation and regeneration and underscore the necessity of context-specific PRF application.

The combination of PRF with MSCs is widely regarded as a promising regenerative strategy as PRF provides both a fibrin-based scaffold and a reservoir of bioactive mediators, including PDGF, TGF-β, and VEGF [45]. Recent preclinical studies have demonstrated synergistic effects of PRF and MSCs in vitro supporting MSC proliferation, and differentiation, as well as in bone and soft tissue regeneration [46,47]. However, most of these studies rely on PRF derived from healthy donors and do not account for trauma-induced alterations in PRF composition.

In contrast to this prevailing assumption, incubation with surgical trauma-activated PRF membranes induced a pronounced suppression of MSC metabolic activity at early time points. Although metabolic activity partially recovered over time—most notably in the surgical trauma-activated PRF group—it remained below medium control levels, suggesting cellular adaptation rather than robust regenerative stimulation. These findings contrast with reports describing proliferative effects of PRF on MSCs and are likely attributable to methodological differences, including PRF processing. In the present study, PRF was processed into a membrane, whereas Wachtel et al. used unprocessed PRF to generate conditioned medium [46]. Furthermore, the use of a dynamic coculture system allowing reciprocal signaling between PRF-resident cells and MSCs was applied. Such bidirectional interactions may generate inhibitory as well as activating feedback loops and more closely reflect in vivo conditions.

At the molecular level, surgical trauma-activated PRF induced transient upregulation of MAPK8 (JNK1) and MAPK14 (p38α), pathways classically associated with cellular stress and inflammatory activation [48]. These kinases are activated by PRF-derived mediators such as PDGF, TGF-β, and IL-1β and exert context-dependent effects on MSC fate. While short-term MAPK activation may support cellular adaptation and survival, sustained activation has been linked to impaired proliferation and differentiation [46,47,49,50]. However, MAPK signaling is also involved in desirable osteogenic differentiation processes, ultimately leading to the expression of osteogenic transcription factors such as RUNX2. For example, p38 and ERK pathways are often associated with pro-osteogenic effects, whereas the role of JNK activation in osteogenesis appears less consistent [51]. The normalization of MAPK expression at later time points supports the concept of inflammatory priming.

Despite the lack of sustained osteogenic differentiation, surgical trauma-activated PRF exerted pronounced pro-angiogenic effects at the early time point. The pro-angiogenic properties of PRF are well documented, and VEGF represents a central regulator of neovascularization [52,53]. Previous studies by our group and others have demonstrated VEGF release from A-PRF. In the present study, VEGF levels in both C-PRF– and T-PRF–MSC cocultures were markedly higher compared to A-PRFalone, analyzed in earlier experiments [19,54], indicating a substantial contribution from MSCs. The increased VEGF release at 24 h in T-PRF group indicates that PRF-derived factors—particularly enriched in surgical trauma-activated PRF—stimulate VEGF synthesis in MSCs. The literature data support the notion that PRF-associated mediators activating MAPK signaling pathways, such as PDGF, or PI3K/Akt signaling, such as IGF-1, promote VEGF expression in MSCs [55,56]. In addition, cytokines such as IL-6 may indirectly enhance VEGF production by amplifying inflammatory and repair-related signaling cascades [57]. Collectively, these findings indicate that surgical trauma-activated PRF preserves, or even enhances, the angiogenic capacity of MSCs.

No clear evidence of early osteogenic activation was observed in this study. Gene expression of RUNX2 and COL1A1 remained unchanged or was reduced compared with controls, contrasting with reports describing pro-osteogenic effects of PRF in longer-term studies [47]. Admittedly, no additional osteogenic parameters were assessed to support this assumption in the present study; therefore, conclusions in this regard remain limited. An important question arising from these findings is why surgical trauma-activated PRF preserved or enhanced angiogenic signaling in MSCs, while probably failing to promote osteogenic gene activity. This apparent divergence reflects the fundamentally different temporal and microenvironmental requirements of angiogenesis and osteogenesis. Angiogenic responses, particularly VEGF induction, are known to be tightly coupled to early inflammatory and stress-related signaling, including MAPK and PI3K/Akt activation, hypoxia-related cues, and cytokine exposure [58]. In contrast, osteogenic differentiation requires a more stable, low-inflammatory microenvironment and sustained activation of lineage-specific transcriptional programs [59,60]. Thus, the inflammatory priming observed here may preferentially shift MSC function toward angiogenic support rather than matrix-producing differentiation. This concept is consistent with the role of early angiogenesis as a prerequisite for later bone formation [61,62].

Moreover, the relatively short observation period may have captured an early, inflammation-dominated phase of MSC adaptation, whereas osteogenic differentiation typically requires prolonged exposure to permissive signals and suppression of inflammatory cues. Together, these findings suggest that trauma-activated PRF promotes an angiogenic, immunomodulatory MSC phenotype that may facilitate early repair but requires subsequent resolution of inflammation to enable osteogenic differentiation.

In parallel, MSCs exhibited signs of compensatory immunomodulation. The delayed increase in IDO-1 secretion suggests an adaptive anti-inflammatory response to PRF-induced immune activation. Given the established role of IDO-1 in suppressing cytotoxic T-cell activity, this mechanism may indirectly support regeneration by limiting immune-mediated tissue damage rather than by directly promoting differentiation [63,64,65]. Similarly, only modest induction of TNFAIP6 (TSG6) gene expression was observed, consistent with a moderate but persistent inflammatory stimulus rather than excessive cytokine release [66].

The patients included in the present study sustained only relatively mild isolated trauma, and the mean interval between injury and surgery was eight days. It can therefore be assumed that the initial trauma-induced systemic inflammatory response had largely subsided by the time of PRF preparation. The magnitude and temporal dynamics of post-traumatic inflammation are closely correlated with injury severity [38]. Previous work by our group has demonstrated that pro-inflammatory cytokines such as IL-6 decline markedly within five days, even after severe polytrauma, and that monocyte function in mildly injured patients returns to levels comparable to healthy controls as early as posttraumatic day two [67,68]. Previous studies suggest that the magnitude of systemic inflammatory responses in isolated extremity trauma and following surgical intervention of the extremities can be comparable [69]. Consequently, the altered biological properties of PRF observed in the present cohort of mildly injured patients are most likely attributable predominantly to the surgical trauma rather than to the preceding traumatic injury itself [70]. Therefore, we consider the term “surgical trauma-activated PRF” as reflecting a clinically relevant state of systemic activation rather than a strictly isolated trauma-specific condition.

From a clinical perspective, our present findings suggest that surgical trauma-activated PRF might act rather as an inflammatory and angiogenic stimulus that may facilitate early phases of repair but might constrain sustained regeneration if inflammation persists. While such properties may be advantageous in acute injury settings requiring rapid vascularization and immune activation, they may be counterproductive in conditions demanding prolonged osteogenic differentiation, such as large bone defects or chronic wounds.

In conclusion, this exploratory study identifies trauma-activated PRF as an inducer of early inflammatory and stress responses in MSCs, accompanied by probably preserved angiogenic but limited osteogenic signaling. Future research should specifically address the standardization of trauma-associated PRF (T-PRF), as donor-related systemic conditions—such as inflammation following trauma—are likely to influence its biological properties. In particular, systematic investigations are needed to determine how variations in centrifugation parameters and inflammatory status affect the cellular and molecular composition of T-PRF. In addition, strategies to modulate the inflammatory profile of T-PRF should be explored, with the aim of optimizing its balance between pro-regenerative and immunomodulatory effects. A deeper understanding of how trauma-induced systemic activation alters PRF functionality may enable the development of more targeted and reproducible therapeutic applications [71,72].

## Figures and Tables

**Table 1:** Genes analyzed by quantitative real-time PCR, corresponding protein names, and NCBI gene identification numbers.

Analyzed Gene	Protein	NCBI ID:
*COL1A1*	Collagen1α	1277
*CXCL10*	CXCL10	3627
*IDO1*	IDO-1	3620
*IL6*	IL-6	3569
*MAPK8*	MAPK-8	5599
*MAP3K14*	MAPK-14	9020
*RUNX2*	RUNX2	860
*TNFAIP6*	TSG-6	7130
*VEGFA*	VEGF-A	7422
*Gapdh*	GAPDH	2597

**Table 2:** Patient characteristics and demographic data.

	Age [Yrs, Mean/SD]	Age Range [Yrs]	Sex % (n)
Healthy volunteers n = 7	38 ± 12	22–58	f: 57% (4) m: 43% (3)
Patients with mono trauma, n = 7	44 ± 18	30–81	f: 43% (3) m: 57% (4)

## Data Availability

The original contributions presented in this study are included in the article/Supplementary Materials. Further inquiries can be directed to the corresponding author.

## Supplementary Materials

The following supporting information can be downloaded at https://www.mdpi.com/article/10.3390/cells15100945/s1, Figure S1: Trilineage differentiation and phenotypic characterisation of the MSC pool. MSC cultivated under control conditions are shown in (A). (B) shows adipogenic differentiated MSC with cytoplasmatic lipid droplets, Alizarin red staining confirmed osteogenic differentiation of MSCs (C) and the dimethylmethylene blue stained pellet culture (D) indicates chondrogenic differentiation. Cultivation time was two weeks for adipogenic and osteogenic differentiation and three weeks for chondrogenic differentiation. (E) and (F) show expression of negative marker CD34 and CD45. In (G) and (H) surface expression of MSC marker CD90 and CD105 is presented, green line = antibody staining, violet histogram = isotype control. All procedures are described in detail in Söhling N, Al Zoghool S, Schätzlein E, Neijhoft J, Oliveira KMC, Leppik L, Ritz U, Dörsam E, Frank J, Marzi I, Blaeser A, Henrich D. In vitro Evaluation of a 20% Bioglass-Containing 3D printable PLA Composite for Bone Tissue Engineering. Int J Bioprint. 2022 Aug 17;8(4):602. doi: 10.18063/ijb.v8i4.602. PMID: 36404794; PMCID: PMC9668481. [43].

## Abbreviations

The following abbreviations are used in this manuscript:

CXCL10	C-X-C motif chemokine ligand 10
DNA	Deoxyribonucleic acid
ELISA	Enzyme-linked immunosorbent assays
H	Hour
IL-1β	Interleukin-1β
IL-6	Interleukin-6
JNK	c-Jun N-terminal kinases
MSC	mesenchymal stromal cells
NK cells	Natural killer cells
PDGF	platelet-derived growth factor
PRF	platelet-rich fibrin
RNA	Ribonucleic acid
RT-PCR	Reverse transcription polymerase chain reaction
TGF-β	transforming growth factor beta
Th cells	T helper cells
TNF-α	Tumor necrosis factor-alpha
VEGF	vascular endothelial growth factor
