# Supplementary material for: The Impact of Surgical Trauma-Activated Platelet-Rich Fibrin on Mesenchymal Stromal Cells In Vitro
Source: Cells. 2026 May 21;15(10):945. doi: 10.3390/cells15100945 (PMC13204544; doi:10.3390/cells15100945)
Supplement: Supplementary file 1 [file cells-15-00945-s001.zip › cells-4278141-supplementary.pdf]

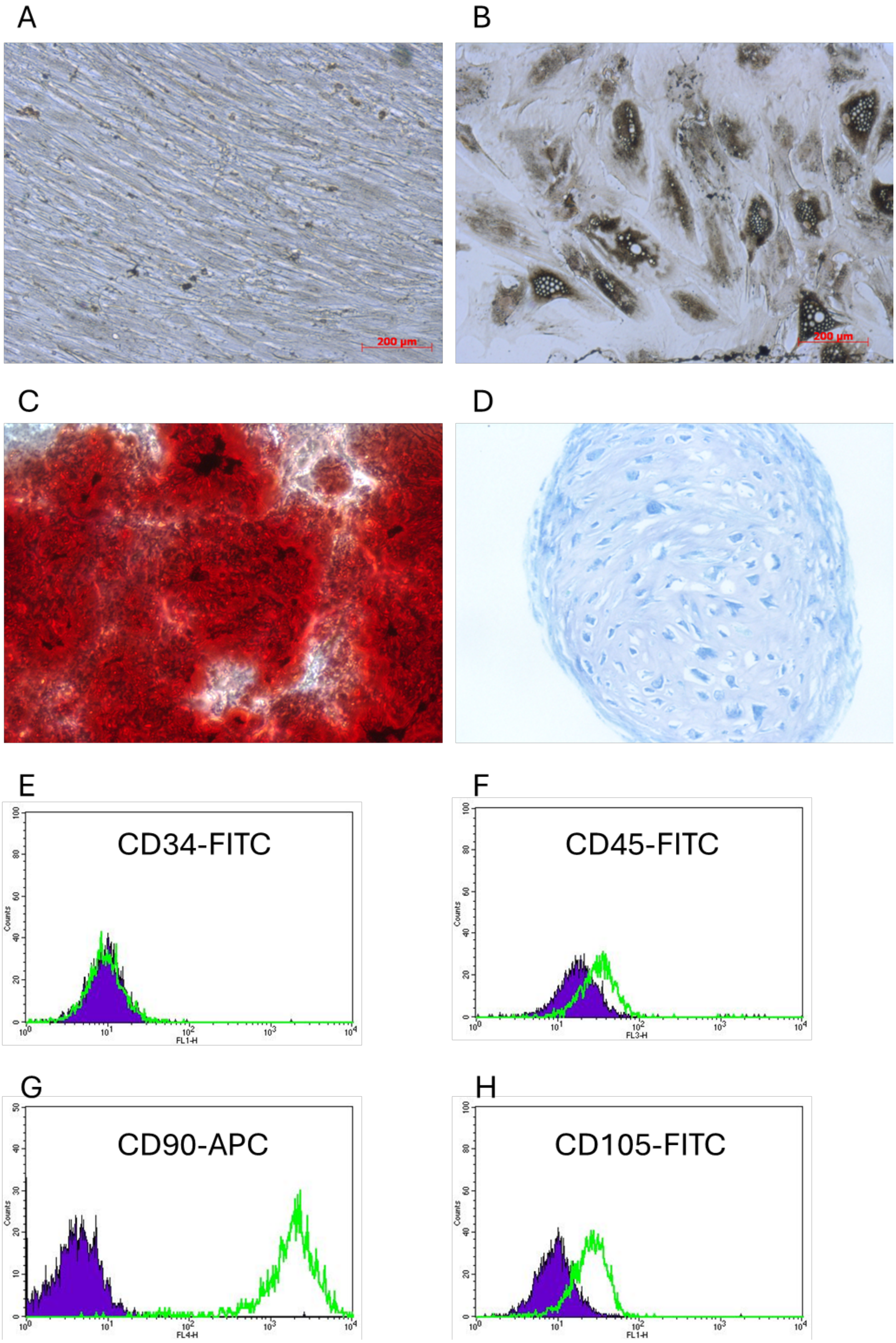
A
B
200 μm
200 μm
C
D
E
CD34-FITC
FL1-H
F
CD45-FITC
FL3-H
G
CD90-APC
FL4-H
H
CD105-FITC
FL1-H

Supplemental Figure S1: Trilineage differentiation and phenotypic characterisation of the MSC pool. MSC cultivated under control conditions are shown in (A). (B) shows adipogenic differentiated MSC with cytoplasmatic lipid droplets, Alizarin red staining confirmed osteogenic differentiation of MSCs (C) and the dimethylmethylene blue stained pellet culture (D) indicates chondrogenic differentiation. Cultivation time was two weeks for adipogenic and osteogenic differentiation and three weeks for chondrogenic differentiation. (E) and (F) show expression of negative marker CD34 and CD45. In (G) and (H) surface expression of MSC marker CD90 and CD105 is presented, green line= antibody staining, violet histogram=isotype control. All procedures are described in detail in Söhling N, Al Zoghool S, Schätzlein E, Neijhoft J, Oliveira KMC, Leppik L, Ritz U, Dörsam E, Frank J, Marzi I, Blaeser A, Henrich D. In vitro Evaluation of a 20% Bioglass-Containing 3D printable PLA Composite for Bone Tissue Engineering. Int J Bioprint. 2022 Aug 17;8(4):602. doi: 10.18063/ijb.v8i4.602. PMID: 36404794; PMCID: PMC9668481.
